# Docking-based modeling of protein-protein interfaces for extensive structural and functional characterization of missense mutations

**DOI:** 10.1371/journal.pone.0183643

**Published:** 2017-08-25

**Authors:** Didier Barradas-Bautista, Juan Fernández-Recio

**Affiliations:** Life Sciences Department, Barcelona Supercomputing Center (BSC), Barcelona, Spain; Koc Universitesi, TURKEY

## Abstract

Next-generation sequencing (NGS) technologies are providing genomic information for an increasing number of healthy individuals and patient populations. In the context of the large amount of generated genomic data that is being generated, understanding the effect of disease-related mutations at molecular level can contribute to close the gap between genotype and phenotype and thus improve prevention, diagnosis or treatment of a pathological condition. In order to fully characterize the effect of a pathological mutation and have useful information for prediction purposes, it is important first to identify whether the mutation is located at a protein-binding interface, and second to understand the effect on the binding affinity of the affected interaction/s. Computational methods, such as protein docking are currently used to complement experimental efforts and could help to build the human structural interactome. Here we have extended the original pyDockNIP method to predict the location of disease-associated nsSNPs at protein-protein interfaces, when there is no available structure for the protein-protein complex. We have applied this approach to the pathological interaction networks of six diseases with low structural data on PPIs. This approach can almost double the number of nsSNPs that can be characterized and identify edgetic effects in many nsSNPs that were previously unknown. This can help to annotate and interpret genomic data from large-scale population studies, and to achieve a better understanding of disease at molecular level.

## Introduction

Next-generation sequencing (NGS) technologies have dramatically lowered gene sequencing costs, and are providing genomic information for an increasing number of healthy individuals and patient populations. To make the most of all these increasingly available genomic data, we need to understand better the link between the genetic information and the phenotype it produces [1]. In this context, the identification and characterization of genetic variants that can be associated to a given disease is the first step in the pursuit of personalized medicine. Approaches like genomic-wide association studies (GWAS) are generating large datasets of genetic variants associated with disorders, which are being deposited in public databases, such as Online Mendelian Inheritance in Man (OMIM) [2], and the database of Genotypes and Phenotypes (dbGaP) [3].

Among the different possible genetic variants found in human populations, non-synonymous single nucleotide polymorphisms (nsSNPs) are small changes in the DNA of an individual that result in a single aminoacid mutation at protein level. Understanding the effect of disease-related mutations at molecular level can contribute to close the gap between genotype and phenotype and thus improve prevention, diagnosis or treatment of a pathological condition. In a first approach, the impact of a mutation in the functional activity of a protein (e.g. an enzyme) can be described in structural terms, because it produces a change in protein folding and stability [4] or it affects a known active or allosteric site. Many structural and biophysical studies have analyzed the effect of a mutation in protein stability, and this effect can be estimated based on computational modeling and calculations [5]. However, in the majority of the cases, proteins are not acting alone, but are forming specific interactions with other biomolecules and are thus part of intricate interaction networks (metabolic pathways, functional complexes, signaling networks, etc.). Large-scale studies at proteomic level have become widely accessible to the community [6–8] and are generating a diverse and increasing amount of data, including protein binding and pathway information [9–11]. This has allowed the computational construction of genome-wide networks, or "interactomes" [12], in which the biological system can be ideally described as a PPI network, where the nodes are proteins and the edges represent interactions between proteins [13].

In this scenario, it will be important to consider the impact of a given mutation in such networks. By gaining a system-wide perspective of protein functions, we can further study which subsets of PPIs are essential in regulating a particular biological process and how genetic variants such as non-synonymous single nucleotide polymorphisms (nsSNPs) affect these PPIs [13,14]. For instance, if a mutation affects the folding or stability of the protein, it will affect and even disrupt all interactions of the mutated protein. On the other hand, if a mutation is located at a specific protein-binding interface, it could affect only some of the interactions of the mutated protein or "edges" in a particular network, which could have functional consequences for such network (so called edgetic effect) [15]. Indeed, alterations on the edges of the interactome are the underlying cause of many disorders [15–17]. Large-scale structural studies show that pathological mutations are enriched on the domains that are involved in protein-protein interactions [18] and confirm that many disease-related mutations are physically located at protein-protein interfaces [17,19,20]. A different study found that OMIM nsSNPs cause small changes in the binding energy of protein interactions [21]. The integration of available 3D structures of proteins complexes with the interaction network analysis can improve our understanding of the functional mechanisms of disease-related mutations [22]. For example, a study combining interaction network analysis, structural data and energetic calculations, found that a significant percentage of the known pathological mutations in the RAS/MAPK pathways affected the binding affinity of some of the interactions in such network [22–24], which provided a general explanation for some of the differences in phenotype.

In addition to improving our knowledge of disease at molecular level, understanding the role of pathological mutations in protein-protein interactions is also of paramount importance for predicting purposes. The functional prediction of nsSNPs that cause amino acid changes in proteins is difficult because they can modify the cellular behavior through different mechanisms, for instance by affecting protein stability, function, or interactions with other proteins and biomolecules [25], as we mentioned above. Thus, for a given mutation found in a patient screening or in population genomic analyses, it would be important to characterize their potential involvement in protein interactions, in order to improve their annotation and/or predict their pathological character by complementing general predictive methods like PolyPhen-2 [26,27], or SIFT [28].

Thus, in order to fully characterize the effect of a pathological mutation at molecular level and have useful information for prediction purposes, it is important first to identify whether the mutation is located at a protein-binding interface, and second to understand the effect on the binding affinity of the affected interaction/s. However, the limited structural data on PPIs make all the above mentioned studies incomplete. The number of protein-protein complexes with their 3D structure deposited in the Protein Data Bank (PDB) [29] is very small. Weak or transient complex structures are particularly difficult to determine by crystallography or NMR, due to technical limitations. There is a growing gap between the number of protein complexes with available experimental structure and the number of interactions that are being discovered. So far the scientific community has available structural data for around half of the non-redundant proteins in human, but only for less than 7% of the estimated human interactome [30]. Computational methods, such as protein docking [31,32] or post-docking analysis [33,34] are currently used to complement existing experimental efforts and could help to build the human structural interactome [35]. However, the main problem for interactomics application is that, for many cases, accurate prediction of a protein-protein structure by docking is still very challenging. Fortunately, the identification of interface residues, based on sequence conservation or physicochemical properties, is more accurate and can be applied at larger scale. Of special importance is the identification of hotspots residues, which are the ones that contribute the most to the binding energy [36]. We previously showed that it is possible to identify interface hot-spots from docking calculations, without needing prior information of the complex structure [37]. Here we have extended the original pyDockNIP method to predict the location of disease-associated nsSNPs at protein-protein interfaces, when there is no available structure for the protein-protein complex. We have applied this approach to the pathological interaction networks of six diseases with low structural data on PPIs. Our method finds 51% of the known interface disease-associated nsSNPs with 62% precision, and predicts a significant number of additional disease-associated nsSNPs (1.5 times the number of known disease nsSNPs based only on the structures) that could be involved in protein-protein interactions.

## Results

### Structural analysis of pathological mutations on protein interaction networks

The general aim of this work is to show how docking-based computational approaches can help characterizing disease-related mutations in PPIs at interactomic scale, where the majority of protein-protein interfaces have no structural data. For this purpose, we focused our analysis on the protein-protein interaction networks of six disease phenotypes for which there was detailed structural information for most of the individual proteins within the network, but low structural coverage of the protein-protein interfaces (see Methods). Table 1 shows the number of proteins associated to each disease according to OMIM that have available structure or a reliable homology-based model (see Methods), as well as the number of proteins and complexes forming the first-layer interaction network and their structural coverage.

**Table 1:** Structural coverage of the disease-related protein interaction networks analyzed in this work.

Phenotype	OMIM code	Associated proteins^a^	Protein interaction networks	
			Proteins^b^	Interactions^c^
**HIGM5**	608106	2 (2, 0)	17 (6, 11)	21 (5, 0)
**LHON**	535000	6 (0, 6)	23 (11, 10)	34 (9, 1)
**CRC**	114500	10 (4, 6)	270 (142, 81)	691 (102, 43)
**MCI**	608446	11 (8, 3)	193 (102, 61)	582 (101, 57)
**HIV-1**	609423	25 (13, 12)	91 (63, 21)	142 (59, 13)
**CMH**	192600	7 (3, 4)	198 (84, 80)	531 (66, 43)
**All six diseases**^d^		61 (30, 31)	729 (361, 249)	1934 (311, 151)

^a^ Number of proteins associated with each disease according to the OMIM database, with available structure or a reliable homology-based model (see Methods). In brackets, the number of proteins with available structure and those with homology-based model, separated by comma (#structures, #models).

^b^ Number of proteins forming the 1st-layer interaction networks of the disease-associated proteins (see Methods). In brackets, number of proteins with available structure or homology-based model, separated by comma.

^c^ Number of interactions in the interaction networks of the proteins associated to each disease. In brackets, protein-protein complexes with available structure or homology-based model, separated by comma.

^d^ Global data for all the selected six diseases, after removing redundant data (union of the different data for the individual diseases).

**Fig 1:**
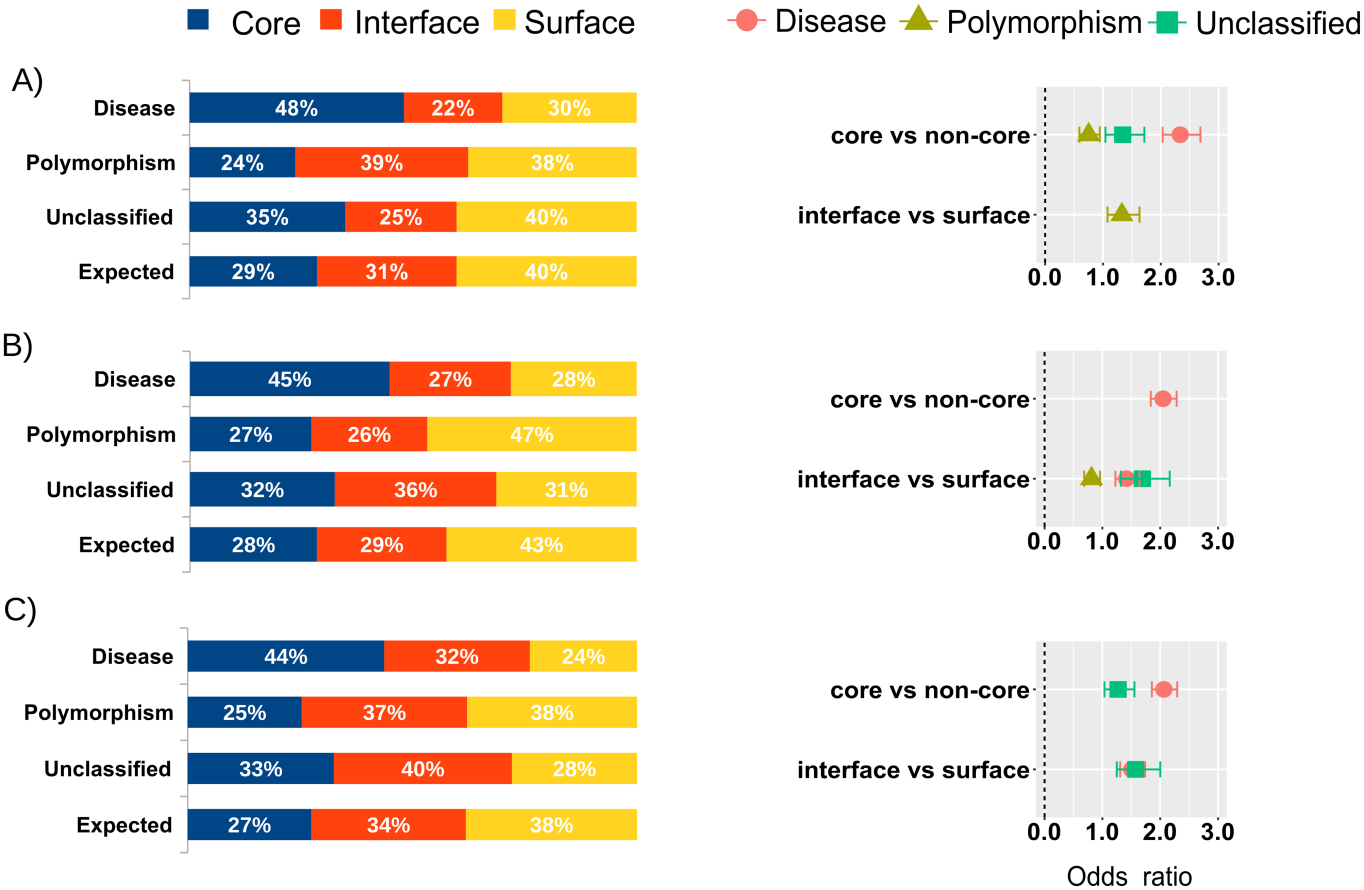
Distribution of nsSNPs in the protein interaction networks of six selected diseases. Distribution of nsSNPs (detailed for disease, polymorphism and unclassified) in the protein interaction networks from the six selected diseases, as classified in core, interface and surface non-interface, with expected distributions as calculated from residue composition, and odds ratios (OR) for the different residue locations and types of nsSNPs, based on (A) structural data; (B) modelled interactions; and (C) combined structural data and modelled interactions. Only significant OR values (*P* < 0.05) are shown.

We first analyzed the distribution of nsSNPs within the protein interaction networks of the six analyzed diseases (see Methods), considering only those protein-protein interactions that had available structure (or a reliable homology model). This structural dataset was formed by 462 protein-protein complexes that had available structure (or a reliable homology-based model), and involved 353 proteins with available structure (experimental or modelled). We found that 258 of these proteins had at least one annotated nsSNP (Table 2). The entire set comprised a total of 1,624 nsSNPs that could be structurally characterized using the complex structures, of which 832 were related to a disease (not necessarily any of the originally analyzed six diseases), 499 were classified as polymorphisms, and 293 were unclassified. Among the structurally mapped disease nsSNPs, 48% are buried, 22% are located at a protein-protein interface, and 30% are found at a non-interacting surface (Fig 1A). We can compare these numbers with the values expected by chance for buried, interface and non-interface residues (29%, 31% and 40%, respectively), as estimated from the residue composition of the studied proteins (see Methods). Thus, the observed/expected (O/E) ratios for buried, interface and non-interface disease nsSNPs are 1.68, 0.70 and 0.75, respectively. The disease nsSNPs are located in buried positions clearly more often than expected by random, which has already been observed in previous studies [17,19]. However, the O/E value for the interface disease nsSNPs obtained here (0.70) is clearly below that reported in previous studies on a large interaction data set (0.96 [17]; an earlier studied found this value to be 1.20, but in that case interface residues were defined exclusively based on distance criteria and could include some buried residues [19]). More interesting is to analyze the preference of a disease nsSNP for being at a protein-protein interface rather that at a non-interacting surface, computed as an odds ratio (OR) (see Methods). Here, we found that disease nsSNPs had similar probability of occurring at protein interfaces than at non-interacting surfaces (OR 0.94) (S1 Table). Again, this value is lower than that previously reported on a large interaction dataset, in which they found a clear preference of disease nsSNPs to be at interface regions rather than non-interacting surfaces (OR 1.35 [17]). The lower preferences found here for the disease nsSNPs to be located at protein-protein interfaces can be explained by the low structural coverage of the protein interactions in the six diseases studied here (which were indeed selected because they had high structural coverage for the individual proteins but low structural coverage on the protein-protein complexes). This shows that the lack of structural data on protein-protein complexes might underestimate the role of many disease nsSNPs involved in protein interactions and can lead to poor characterization of the effect of these mutations in the network topology.

**Table 2:** Detailed analysis of the location of nsSNPs in the disease-related protein interaction networks, based on complex structures and modelled interactions.

	Structural data	Docking-based models	Structural data & docking-based models
**Total number of proteins**	353	583	603
**Proteins with known nsSNP**	258	411	424
**Total residues**			
Number of residues	76168	189629	199846
Core residues	21710	53849	54936
Interface residues	23779	55031	68768
Non-interacting surface residues	30679	80749	76142
Hot-spot residues	5918	11839	16449
Hot-spot residues at interface	3673	11839	14459
**Total nsSNPs**			
Number of nsSNPs	1624	2615	2786
Disease	832	1363	1438
Polymorphism	499	851	899
Unclassified	293	401	449
**Non-interacting surface nsSNPs**			
Disease	250	384	343
Polymorphism	188	399	340
Unclassified	118	126	125
**Core nsSNPs**			
Disease	399	609	629
Polymorphism	118	231	228
Unclassified	102	130	146
**Interface nsSNPs**			
Disease	183	370	466
Polymorphism	193	221	331
Unclassified	73	145	178
**Interface hot-spot nsSNPs**			
Disease	33	74	109
Polymorphism	46	35	76
Unclassified	17	47	61

Thus, it remains to be seen whether having more structural data on the protein interactions for these six diseases analyzed here could improve the structural and functional characterization of known disease-related nsSNPs. The following section will explore computational ways to extend the structural characterization of protein interaction networks.

### Prediction of interface residues by docking

**Fig 2:**
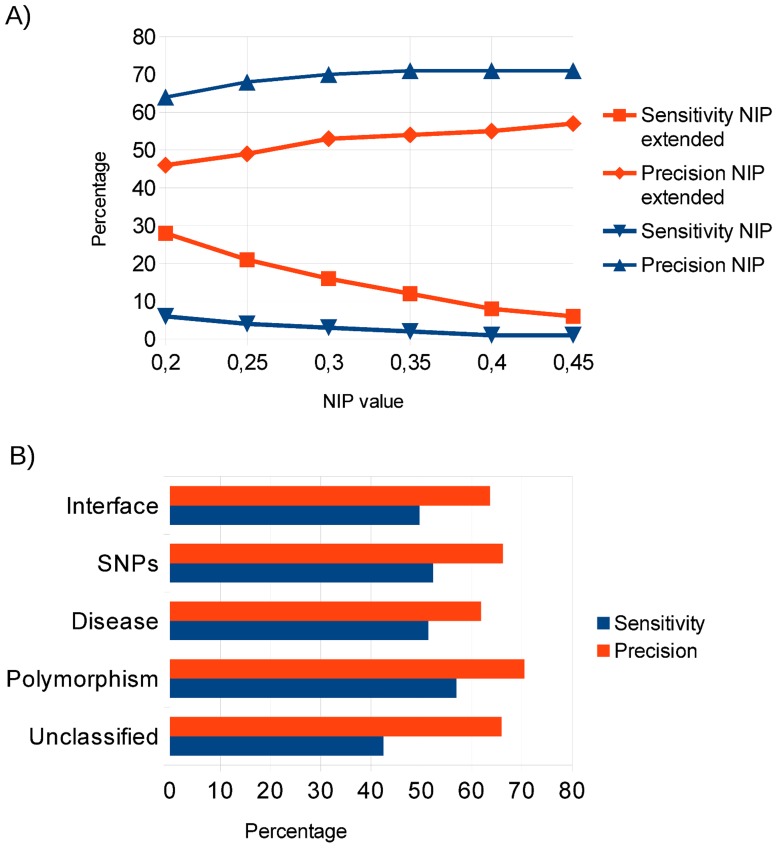
Prediction of interface residues and nsSNPs. (A) Prediction success (sensitivity and precision) of interface residues using pyDockNIP (alone or extended with neighbor residues) on the proteins of the protein-protein docking benchmark 4.0, according to NIP cutoff value. (B) Interface and nsSNPs predictions using the extended pyDockNIP predictions on the proteins of the structural interaction networks from the six selected diseases. The nsSNPs predictions are detailed for interface disease-related, polymorphism and unclassified nsSNPs.

The main goal of this work is to explore computational ways of characterizing pathological mutations possibly involved in protein-protein interactions for which there is no available structural data. We previously found that energy-based protein docking can be efficiently applied to identify interface and hot-spot residues in protein-protein complexes [37]. Basically, from the resulting docking poses, we obtained a normalized interface propensity (NIP) per residue, which describes how often a given residue is involved in the 100 lowest-energy docking interfaces (see Methods). This approach was implemented in the pyDockNIP module within our docking protocol pyDock [33]. We have evaluated the predictive capabilities of this method at different NIP cutoff values, on the protein-protein docking benchmark 4.0, and the results (Fig 2A) confirm that this method can predict interface residues with high precision (65–70%), but very low sensitivity (less than 10%). This sensitivity level is too low for its applicability at large protein interaction networks, given that the majority of pathological mutations involved in protein interfaces would not be detected. In order to improve its applicability, we have extended the predicted interface patches by including residues in the vicinity of the originally predicted ones (see Methods). This strategy showed a better trade-off between precision and sensitivity, with improved sensitivity up to 28%, at the expense of precision (Fig 2A).

As an additional test, we applied the extended interface predictions to the structural interaction networks of six selected diseases, as above mentioned, containing 462 protein-protein interactions for which the complex structure is available or can be modelled based on a homologous template, which involved 353 proteins with available structure (or a reliable homology-based model). Some of the proteins in this dataset had more than one binding partner, so we considered as interface residues those that are involved in any of the possible interactions. As a consequence, 44% of the surface protein residues were observed to be located at a protein-protein interface (Table 2). Then computational docking was run on the separated complex components of the 449 protein-protein complexes, being them either x-ray structures or homology-based models, and the extended interface predictions were compared to the real interface residues. The predictions yielded a precision of 64%, with a sensitivity of 50% (Fig 2B). This improvement in the predictive success rates with respect to the results in the protein-protein docking benchmark might be due to the fact that many of the proteins in the disease-associated interaction networks showed several binding partners, and thus the observed proportion of interface residues in that set (44%) was larger than in the docking benchmark (23%). To estimate the random accuracy, we randomly selected 44% of the surface residues as random interface predictions (to keep the same proportion as in the real interfaces), and this approach showed 43% precision and 36% sensitivity for the prediction of interface residues in the structural interaction networks. As an additional test, we selected a small set of 28 proteins that had only one known interacting partner in the structural interaction networks (i.e. we disregarded proteins with multiple interactions), and we docked all these proteins with randomly chosen proteins that were different from their known partners. Using these random docking pairs, our extended interface predictions showed 46% precision, and 23% sensitivity for the prediction of interface residues. This shows that the docking-based interface predictions proposed in this work achieves predictive success rates well above random, for which using the specific partner/s in docking is critical.

### Docking-based interface prediction can help to improve nsSNP characterization

We then tested the docking-based extended interface predictions on all the nsSNPs found in the structural interaction networks of the six selected diseases. We applied this methodology to the identification of interface nsSNPs, and the predictive success rates were similar to those of the interface predictions (Fig 2B). As an additional test, we focused on the disease-related nsSNPs within the same structural interaction networks. Thus, we applied our docking-based method to the 832 disease-related nsSNPs in our structural dataset in order to predict whether they were located at interfaces. When compared with the 183 disease-related nsSNPs that were actually located at interfaces in our structural dataset, the predictions showed very similar numbers in precision (62%) and sensitivity (51%) to those for the interface predictions (Fig 2B). When applied to other types of nsSNPs, the prediction success rates were also similar, except for the "unclassified" nsSNPs, for which sensitivity is slightly lower (Fig 2B). In general, the above results show that docking-based predictions can identify with reasonable precision when a given nsSNP is located at a protein-protein interface, independently on whether such nsSNP is associated to a disease or no. This provides a valuable resource to characterize nsSNPs in cases with no structural information on the potential protein-protein interactions.

### Identification of interface nsSNPs in complexes with no available structure

The above described protein interaction networks for the six selected diseases contained 1,472 interactions for which there is no available structure. They involved as many as 3,323 nsSNPs that could not be structurally mapped in such interactions. Some of these nsSNPs might have been considered in the previous analysis of the structural interaction network dataset, simply because they were involved in other complexes with available structure, but they still lacked information for all the other interactions with no available structure. In 1,055 of these interactions, the interacting subunits had available structure or could be easily modelled by homology, which made them suitable for docking calculations. In total, there were 583 proteins with structure or easily modelled by homology (Table 2). We ran docking simulations on these interactions to predict interface residues, and then used this information to identify nsSNPs located at protein-protein interfaces. Some of the interacting proteins have different PDB structures corresponding to different parts of the protein, in which case we used all of these structures independently in docking. For instance, in the interaction between the oncogene RAF1 and the heat shock protein HSP90AA1, there are five different PDB structures associated to RAF1, covering different zones of the protein, and two different PDB structures associated to HSP90AA1. Such discontinuous structural coverage for these proteins makes that the modeling of this interaction alone needs 10 independent docking simulations. As a consequence, we run a total of 8,920 docking simulations, and as many of 2,615 nsSNPs could be characterized in 1,055 modelled protein-protein complexes. Within these nsSNPs, we found 1,363 disease-related, 851 polymorphisms, and 401 unclassified. Among the docking-based characterized disease nsSNPs, 45% were buried, 27% were located at a protein-protein interface, and 28% at a non-interacting region (Fig 1B). According to the residue composition of the studied proteins in the docking predictions, the values expected by chance for buried, interface and non-interface nsSNPs are 28%, 29%, and 43%, respectively. Incidentally, these interface/non-interface residue composition values in the docking predictions show that the predicted interfaces in these interaction networks are similar in size to the real ones. This is an additional advantage of the extended interface predictions over the original NIP-based method, which provided much smaller interfaces. Thus, the O/E ratios for buried, interface and non-interface disease nsSNPs are 1.57, 0.94 and 0.66, respectively. These numbers are virtually the same as those found in previous studies on larger interaction sets (1.58, 0.96, and 0.71, respectively [17]). Based on the modelled interactions, disease nsSNPs have clear preference for being at protein-protein interfaces as compared with non-interacting surfaces (OR 1.42), also in line with previous studies (OR 1.35 [17]). This shows that modeling interaction networks by docking has the capability of extending the characterization of nsSNPs in cases with no available structural data.

### Integrated experimental and computational characterization of protein interaction networks

Then, we combined the results of the structural dataset with the modelled interactions for the protein interaction networks of the six selected diseases. In this way, we had structural or modelled data for 1,517 protein-protein interactions, involving proteins that harbored a total of 2,786 nsSNPs. They contained 1,438 disease-related, 899 polymorphisms and 449 unclassified nsSNPs. Among the characterized disease-related nsSNPs, 44% were buried, 32% were located at interfaces, and 24% at non-interacting regions (Fig 1C). According to the residue composition of the structurally characterized and modelled proteins, the values expected by chance for buried, interface and non-interface residues are 27%, 34%, and 38%, respectively. Thus, the O/E ratios for buried, interface and non-interface disease nsSNPs are 1.59, 0.94 and 0.63, respectively (similar to previous studies [17]). This indicates an even clearer preference of the disease nsSNPs for being at interfaces rather than at non-interacting regions (OR 1.51), a preference that could not be detected before using only structural data due to the limited number of available complex structures for these selected diseases. Indeed, based only on the available structural data, 183 disease nsSNPs were found at protein interfaces, while 250 were found at non-interacting surfaces. When data from docking was included, as many as 112 of these 250 nsSNPs (45%) were actually found at protein-protein interfaces. This clearly shows that the combination of experimental and computational information can help to improve the structural characterization of protein interaction networks and the identification of nsSNPs involved in interactions, which could not be previously found due to the lack of structural data.

Interestingly, the disease-related nsSNPs that are estimated to be interacting hot-spots according to the docking-based predictions (interface residues with NIP > 0.2) show an O/E ratio of 1.05, and a clear preference over the non-interacting regions (OR 1.68), similar to that previously reported for interface core disease nsSNPs vs. non-interacting regions (OR 1.72 [17]).

For the interaction networks of the six selected diseases, on top of the 183 disease-related nsSNPs that could be structurally mapped at protein-protein interfaces, we found 283 additional disease-related nsSNPs that were predicted to be at an interface based on the docking models. We should note that some of the nsSNPs residues predicted as interface from the docking models could have been already defined as interface from the complex structures, because they were involved in other interactions for which there was available structure, and this is why last column in Table 2 is not simply the sum of the two previous columns. Considering all the available structural and docking-based data, we found a total of 109 interface disease-related nsSNPs that were also characterized as hot-spots, and which are likely to have a significant edgetic effect (see Discussion).

### Docking-based characterization of pathological mutations in the RAS/MAPK pathway

We used our interface prediction method to extend the characterization of nsSNPs in other protein interaction networks. A recent comprehensive study on pathological mutations involved in cancer and RASopathies in proteins of the RAS/MAPK pathway showed that around 20% of the structurally-mapped pathological mutations were predicted to have a direct effect on protein-protein or domain-domain interfaces [24]. However, for over 30% of the mutations that could be mapped at a protein structure, they could not find any structural or energetic reason that might explain their pathological character. The majority of these mutations were located at the protein surface, and the authors proposed that they might be involved in protein interactions for which there is no sufficient structural data. Some of the mutations could be located at a known protein-protein interface but perhaps do not have any impact on the binding affinity [21], while they could actually affect other protein-protein interactions with no available structural data [38,39]. Therefore, we aimed to complete the interface structural and energetics data of this protein interaction network with our computational approach, to explore whether this can help characterizing some of these "unexplained" mutations. We used the first-degree neighbors from Interactome3D server [30] to construct the network for the 15 proteins analyzed in the mentioned study [24].

**Fig 3:**
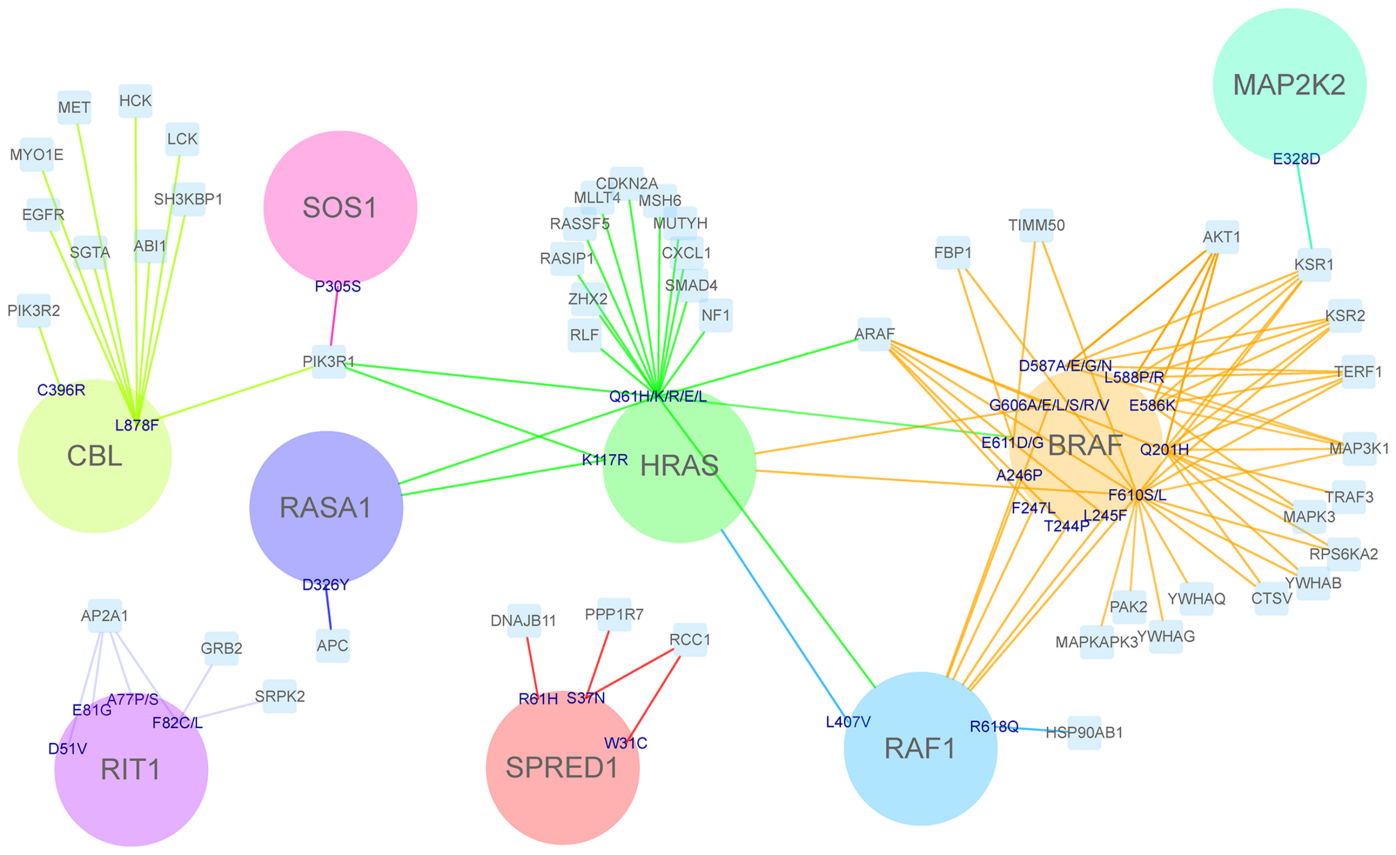
Structurally unexplained pathological mutations of the RAS/MAPK pathway that are predicted to be involved at protein-protein interfaces. Proteins of the RAS/MAPK pathway are represented as circles, showing pathological mutations that were not previously characterized due to the lack of structural data, but that have been predicted here to be binding hot-spots when docking with specific protein partners from this pathway (circles) or from the first-degree interaction network (in cyan squares). These docking partners thus represent proteins whose interaction is predicted to be affected by the mutation to which they are linked. Thus, all edges here correspond to interface predictions from docking.

**Fig 4:**
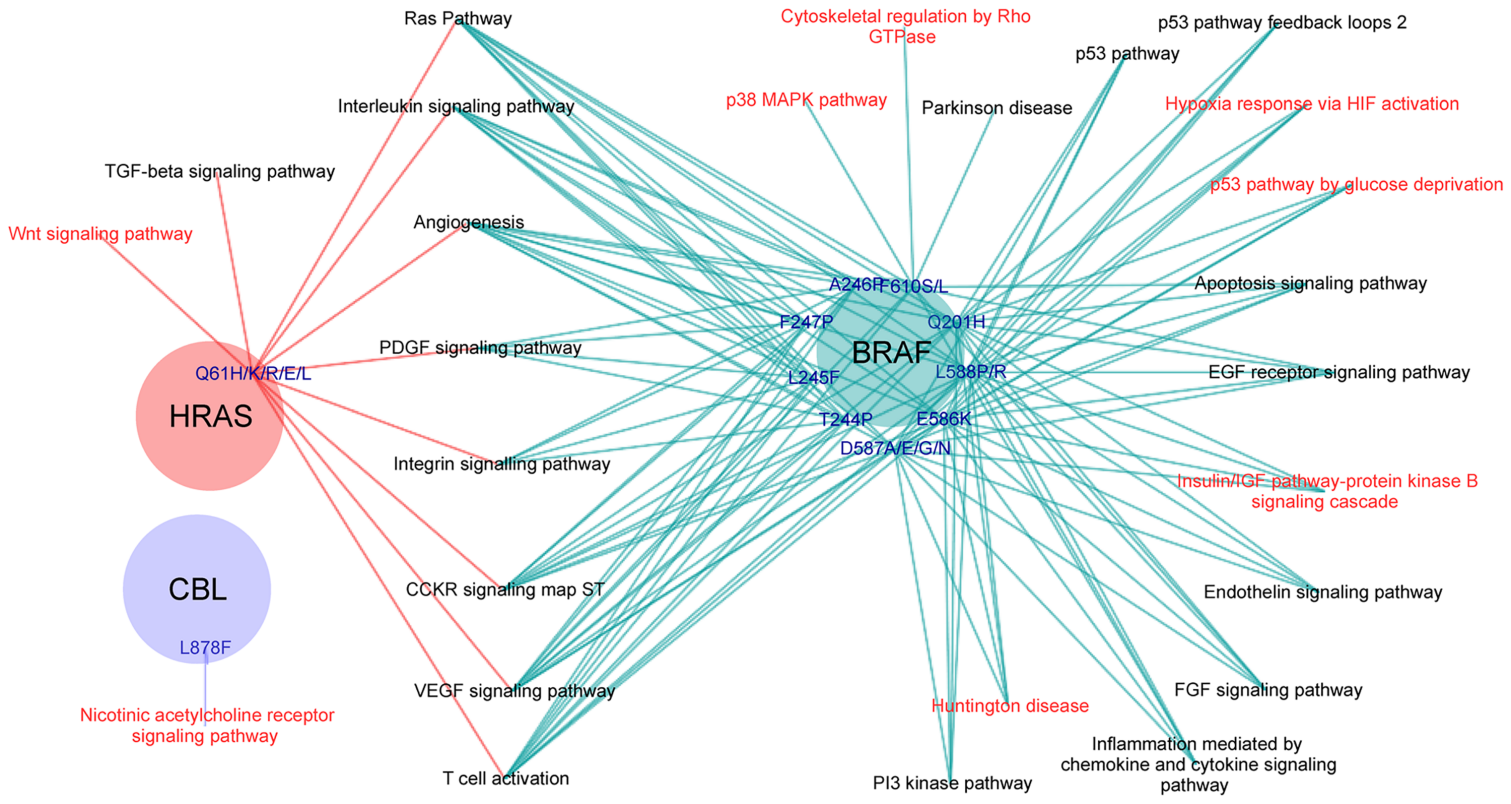
Pathways affected by pathological mutations in RAS/MAPK proteins predicted to be at binding hot-spots. Proteins of the RAS/MAPK pathway are shown as colored circles, showing pathological mutations that were not previously characterized due to the lack of structural data, but that have been predicted here to be binding hot-spots for docking partner proteins involved in other pathways (linked to the corresponding mutation). Pathways shown in red are those that could not have been found using only available structural data.

The complete interaction network involved a total of 236 proteins, 234 of them with available structure, and 482 protein-protein interactions (300 of them without structural information). We performed 1,893 docking calculations on those protein interactions with no available structure, in order to identify the interface and hot-spot residues. From the 208 pathological mutations that were unexplained in the mentioned study [24], we found 95 mutations (in 59 residues of 11 proteins) that were predicted to be at a protein-protein interface based on the docking calculations. That is, interface predictions based on docking calculations helped to rationalize almost half of the unexplained mutations. Among them, we found 44 pathological mutations (in 29 residues of 9 proteins) that were predicted to be located at a protein-protein binding hot-spot residue (Fig 3). These nine proteins play a significant role in the RAS pathway, and are found to interact with several other signaling proteins. Cross pathway connectivity among signaling proteins is a network property that is related to the robustness or fragility of cell functions [39]. Therefore, mutations located at protein-protein interfaces in these nine proteins could not only affect the RAS pathway but also other pathways. Fig 4 shows the pathways involving proteins whose interaction is affected by the pathological mutations predicted to be located at a binding hot-spot. We found the most affected pathways are related to the vascular system formation and activation of immune cells. The VEGF, PDGF, FGF and interleukin signaling pathways are closely involved in cell proliferation, differentiation and angiogenesis, all of them highly relevant in the development of cancer.

## Discussion

### Linking structural information to phenotypes

Structural characterization of nsSNPs and their involvement in protein-protein interfaces is a starting point to understand complex diseases, for which databases like dSysMap [20] are valuable resources. However, a major problem is the limited structural data available for protein-protein complexes, and as a consequence, only a fraction of all possible nsSNPs can be accurately located at the interfaces. In this work, we have used docking models to characterize nsSNPs that are likely to be involved in protein-protein interactions. To test this approach, we have selected six complex diseases in which their associated proteins are involved in protein-protein interactions for which there is little structural data.

The first difficulty we encountered in this analysis was the availability of data. The task of finding all the coding protein genes to construct the protein interaction network of a complex disorder is not trivial, as there are different sources of data for nsSNPs (e.g. *humsavar*) and disorder genes (e.g. OMIM) that are not always fully consistent. Indeed, the gene map file used here from OMIM had 3,012 disease phenotypes, while the version of *humsavar* used in this work has 2,727 phenotypes with assigned nsSNPs. This means that there could be protein coding genes associated to a disease phenotype, which do not have any described nsSNP. An example of this was the phenotype MCI (susceptibility to myocardial infarction) [MIM: 608446]. This phenotype is not considered in databases like dSySmap, because all coding protein genes that have been reportedly associated to the disease harbor mutations for other diseases, and thus no nsSNPs can be found associated with this MIM code in *humsavar* (Table 1). Therefore, a specific analysis of this phenotype using only the nsSNPs annotated in *humsavar* is not realistic. When we analyzed the interaction network of the proteins associated to this disease, including all nsSNPs associated to any other diseases, we found a strong preference of these nsSNPs to be at an interface rather than in non-interacting regions (OR 1.52, *P*-value < 0.005). The involvement of different nsSNPs causing other diseases in the protein-protein interfaces of this interaction network is indicative of a complex genotype-to-phenotype relationship, which is probably masking the nsSNPs linked to this specific MCI phenotype.

**Fig 5:**
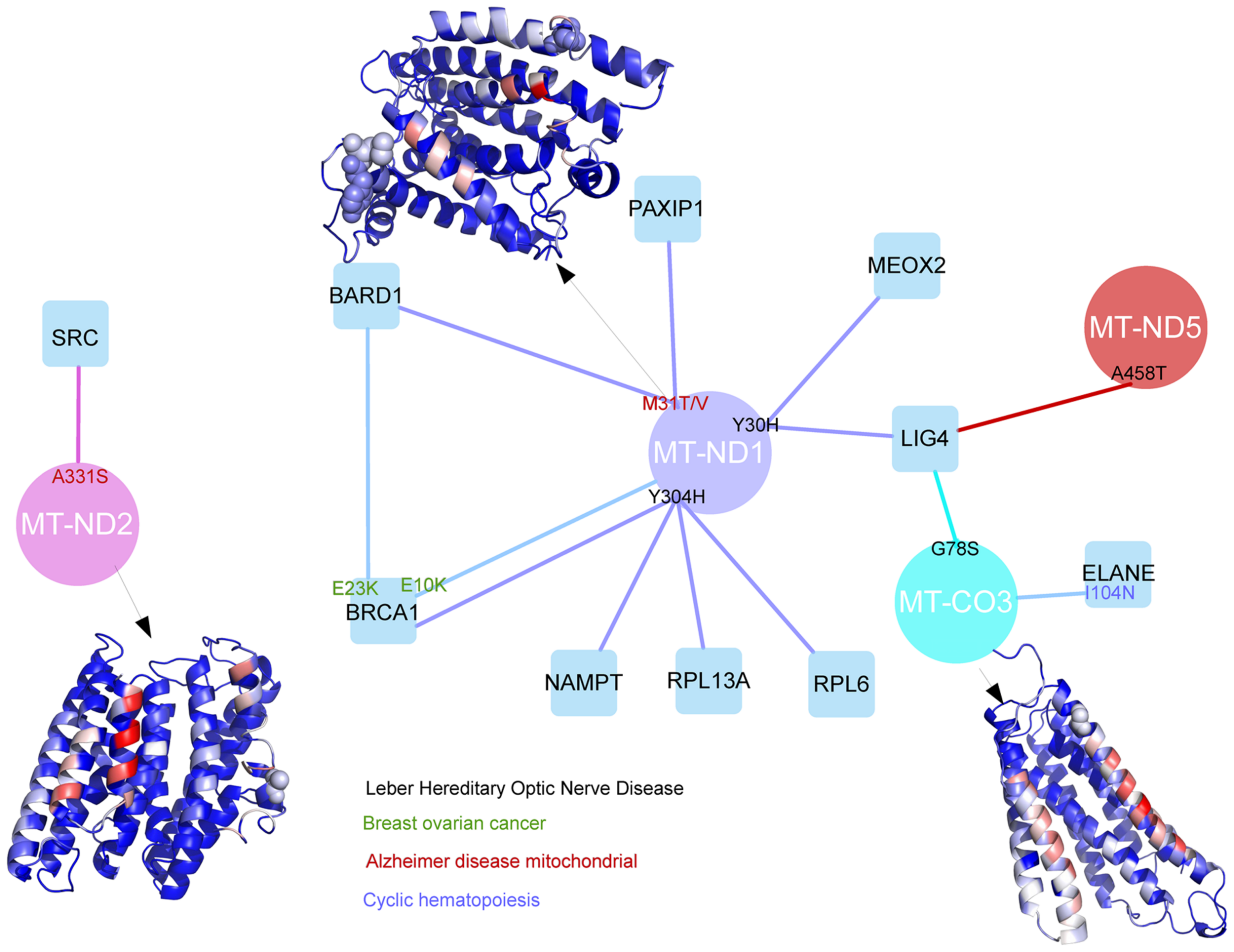
LHON interaction network with disease-related nsSNPs located at modeled protein-protein interfaces. Proteins associated with LHON pathology (circles) and their modeled interactions (edges) with other proteins of the network (squares), showing the disease nsSNPs (for LHON and other pathologies) that are located at the modelled protein-protein interfaces. The homology-modeled structures for selected proteins are shown in ribbon, with disease nsSNPs in CPK representation, and all residues colored according to their NIP value (in red NIP > 0.2; in blue NIP < 0.0).

Due to the limited availability of structural data, in phenotypes like the Leber hereditary optic neuropathy (LHON) [MIM:535000], a rare mitochondrial disease, not a single LHON nsSNP could be structurally mapped at a protein-protein interface, since there were no available structures for the protein-protein interactions associated to this disease (except for the self-interactions, i.e. homocomplexes). Fortunately, we were able to model most of the protein complexes of the LHON interaction network by means of computational docking. In this way, we identified 4 LHON disease-related nsSNPs at protein-protein interfaces, involving 3 proteins (MT_CO3, MT-ND1, and MT-ND5) that are part of the respiratory chain (Fig 5). One of the proteins, MT_CO3 (UniProt P00414), is part of the complex IV assembly of the cytochrome oxidase c, which is the terminal member of the respiratory chain of the mitochondria. The other two affected proteins are components of the NADH-ubiquinone oxidoreductase complex, which is key to the catalytic function of the respiratory chain. We could only analyze part of the chain 1 (MT-ND1, UniProt P03886) and chain 5 (MT-ND5, UniProt P03915). We could also characterize additional nsSNPs in the LHON interaction network related to other diseases like Alzheimer and Breast-ovarian cancer (Fig 5). For instance, MT-ND1 and MT-ND2 harbor additional nsSNPs at the interface that are also linked to other diseases, such as Alzheimer's disease (MIM 502500) and MELAS syndrome (MIM 540000). MT-ND1 and, especially, MT-ND5 proteins are involved in the recognition of BCRT domains. The nsSNPs that we found located in the interface might be very specific for this LHON disorder, probably altering the recognition of such domains. We also found other elements of the respiratory chain affected by nsSNPs at an interface zone, which were described to cause other mitochondrial related disorders. For example, the protein ELANE (P08246), a mitochondrial elastase, is involved in two different diseases, cyclic hematopoiesis (CH; MIM 162800) and severe congenital neutropenia 1 (SCN1; MIM 202700). Interestingly, the nsSNP I104N, which is known to play a role in causing CH, is predicted here to be located at a protein-protein interface.

### Prediction of edgetic effects of SNPs affecting specific pathways

**Fig 6:**
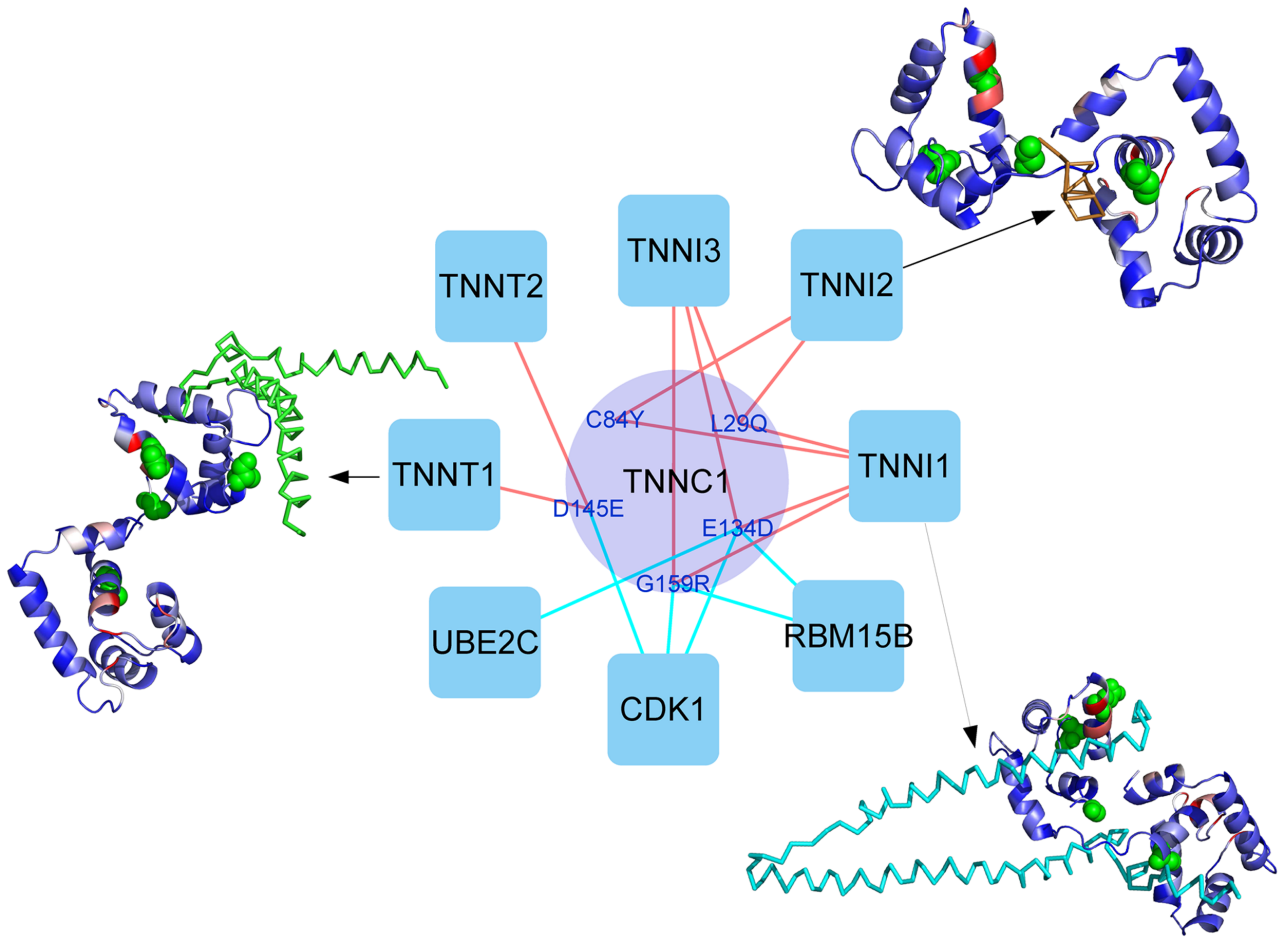
Effect of nsSNPs in TNNC1 interaction network based on complex structures and modelled interactions. Analysis of TNNC1 interaction network by combining structural data and docking models can identify different nsSNPs that could affect the interaction with different proteins. Protein-protein interactions with available structure are represented as red edges. Modelled interactions are represented as cyan edges. Selected protein-protein complex structures are shown, with residue color coding for the predicted NIP values as in Fig 5.

Crosstalk in cellular pathways provides the cell with a robust network of interactions to respond to stimulus. The description of these pathway crosstalk events at molecular level and the mutations that may affect them would open multiple applications in biomedicine, from understanding the homeostatic response of a given drug in a particular population to discovering new personalized scenarios for drug repurposing [40]. A recently reported interaction perturbation profiling of missense mutations across a broad spectrum of human disorders suggests that around one third of disease mutations have edgetic effects [41]. The same study shows that different mutations in the same gene may produce different interaction profiles and, as a consequence, distinct disease phenotypes [41]. However, understanding the possible edgetic effect of hundreds of thousands of mutations arising from genome-wide association studies and gene sequencing efforts is being hampered by our currently limited structural knowledge of protein interactions. The structural characterization of missense mutations by combining complex structures and docking predictions, as shown in this work, can be essential to achieve this understanding at interactomic level. As an example, structural analysis of the TNNC1 interaction network in MHC phenotype (Fig 6) shows that different nsSNPs could affect the interaction with different proteins. Indeed, mutations affecting TNNT1 binding are in different region than those affecting TNNI1 and TNNI2. Docking-based predictions can help to understand the structural role of additional nsSNPs that are involved in interactions for which there is no available structural data. For instance, based on the docking models, CDK1 binding has been found to be affected by TNNC1 nsSNPs D145E, G159R and E134D; UBE2C binding is found to be affected by E134D; and RBM15B binding is found to be affected by G159R and E134D (Fig 6).

In the case of RASopathies, where several of the network nodes are important interaction hubs, a given disease-associated nsSNP at the interface region might have an edgetic effect by affecting certain specific pathways but not others. We found that many of the disease-related nsSNPs that could not be explained in a previous study [24] were located at the docking extended interfaces, thus affecting around 50 protein partners that were involved in 38 different pathways. Moreover, some of these disease-related nsSNPs were located at hot-spot residues, which were found to affect 26 different pathways (Fig 4). As much as 25 of these pathways are mediated by interaction partners of BRAF and HRAS. The remaining one, the nicotinic acetylcholine receptor signaling pathway, was affected by a pathological mutation in CBL. In total, there are 8 pathways affected by the mutations at the predicted hot-spots that would have not been identified based only on the available structural data (Fig 4). According to our hotspot prediction, the pathways that are involving a larger number of proteins whose interaction was predicted to be affected by pathological mutations are the RAS pathway, VEGF signaling pathway, T cell activation and angiogenesis. All of these pathways involve interaction partners of both BRAF and HRAS proteins.

### Conclusions and future perspectives

We have presented here a procedure to improve the characterization of genomic variants involved in protein-protein interactions, especially in cases with low or limited structural information on the binding complexes. The application of interface and hotspot predictions based on docking simulations can extend the structural knowledge of protein-protein interfaces and estimate the role of nsSNPs regarding the interaction with other proteins. We have applied this to selected protein interaction networks for disease in which little structural data for the protein complexes were available. This approach can almost double the number of nsSNPs that can be characterized and identify edgetic effect of many nsSNPs that were previously unknown. In summary, this procedure overcome current limitations of complex structures and can help to understand the structural and functional role of genomic variants involved in protein-protein interactions, and their edgetic effect on specific protein interaction networks within a given disease. Future research will focus on improvement the structural modeling of protein interfaces by novel scoring methodologies, integration with template-based docking, or optimized flexibility treatment. The will help to annotate and interpret genomic data from large-scale population studies, and to achieve a better understanding of disease at molecular level.

## Methods

### Disease-associated proteins and interaction networks

Genes associated with different diseases were obtained from the OMIM database [2]. Thus, 2,394 distinct proteins (based on UniProt ID) were found to be associated to a total of 3,012 disease phenotypes (based on OMIM ID) (S2 Table). From this, pathological interaction networks were built by selecting the first layer interaction partners for proteins associated to each disease using the Interactome3D server [30], which contains only protein-protein interactions for which there is reliable evidence that they are binary. Only disease-associated proteins with available structure (or reliable homology-based model in Interactome3D [30]) were used as seeds to build the interaction networks, in order to be able to map known nsSNPs variants on them (see below). Structural data (experimental or modeled) for the proteins and complexes in each interaction network were also retrieved from Interactome3D [30]. When several structures or models were found for a given case, we selected the ones with the highest coverage and/or sequence identity from the proteins.dat file of the database. In some cases, we found different structures for the same protein (UniProt code), corresponding to different parts of the protein, so they were independently used for the different analyses in this work.

### Statistical analysis of nsSNPs on disease-associated protein interaction networks

In this work, we selected six disease phenotypes for which there were detailed structural information for most of the individual proteins within the network, but low structural coverage of the protein-protein interfaces (Table 1): Hyper-IgM syndrome 5 (HIGM5); Leber hereditary optic neuropathy (LHON); Colorectal cancer (CRC); Susceptibility to myocardial infarction (MCI); Susceptibility to HIV type 1 (HIV-1); and CardioMyopathy Hypertrophic variants 1 to 15 (CMH). S3 Table shows the proteins associated to these six selected diseases (based on the OMIM database). S4 Table shows the proteins contained in the first layer interaction networks of the six selected diseases, and their structural coverage (based on Interactome3D). S5 Table shows the complexes forming the first-layer interaction network of the six selected diseases, and their structural coverage (based on Interactome3D).

The nsSNPs variants for each gene in the protein structural interaction networks associated to the six selected disease phenotypes were obtained from the *humsavar*.*txt* file (release 2014_06 of June 11th, 2014; downloaded from www.uniprot.org) and mapped to the corresponding protein structure (S6 Table). For this, the human sequences with all the variants were downloaded in a FASTA format from the UniProt web page [42]. Then, the sequence and numbering of the PDB files in our dataset were extracted and aligned with the corresponding FASTA sequence when the numbering was incorrect or shifted. The nsSNPs were classified, according to the *humsavar*.*txt* file, as: i) disease-associated, when the mutation is known to cause a disorder; ii) polymorphism, when the mutation is believed to be a neutral mutation; and iii) unclassified, when the mutation is detected in one or few patients, but showed low statistical significance due the limited size of the sample.

Residues in the protein structures were classified as core, interface and non-interacting surface according to the available structural data on the known protein-protein complexes (S6 Table). Core residues were those with relative ASA < 0.1 (relative ASA is the ASA value for a given residue over the ASA reference value of the corresponding residue type). Then, exposed residues (relative ASA > 0.1) were classified as interface residues if any of their atoms are found within 10 Å from another atom from a partner protein. The remaining residues are classified as non-interface surface. When multiple structures exist for a single protein, to avoid ambiguous classifications for the same residue we used for classification the average of relative ASA (rASA) for the residue in each of the structures. In a few cases, the same residue could be defined as core based on the complex structures and as exposed based on the docking predictions (in those interactions with no complex structure, see next section), or vice versa, depending on which structures are used in each case for the average rASA calculations.

The observed/expected (O/E) ratios for the distribution of nsSNPs in the above mentioned protein regions (core, interface and non-interacting surface) were calculated as the observed fraction of nsSNPs found in each protein region over the fraction of nsSNPs expected by chance in each protein region. The latter was estimated from the fraction of total residues found in each protein region in all analyzed proteins.

The preference of a nsSNP for being in a given protein region *i* rather that in a region *j* was computed as an odds ratio (OR), as previously described [19]:

$$OR_{ij}=\frac{P_{i}/\left(\text{1}-P_{i}\right)}{P_{j}/\left(\text{1}-P_{j}\right)}$$ (1)

where *P*_*i*_ is the probability of observing a nsSNP of a given type in protein region *i*, and is computed as:

$$P_{i}=\frac{n_{i}}{N_{i}}$$ (2)

where *n*_*i*_ is the number of nsSNPs of a given type observed in protein region *i*, and *N*_*i*_ is the total number of residues in protein region *i* in all the analyzed proteins. The statistical significance of the OR values were estimated by a two-tailed *P*-value, as previously described [19], using the statistical packages in R (version 3.1.1).

### Prediction of extended interface patches by pyDockNIP

We have developed a new version of the pyDockNIP method for predicting interface residues in a given protein-protein complex, as follows. Docking simulations were run with FTDock [43] to generate 10,000 rigid-body docking poses, which were rescored by pyDock [33] energy-based function composed of van der Waals, electrostatics and solvation energy terms. From the docking results, normalized interface propensity (NIP) values per residue were calculated with the built-in pyDockNIP module [37]. Basically, for each residue, the averaged buried surface (ABS) was calculated as the relative difference between its accessible surface area (ASA) in the unbound structure and the average ASA of that residue in the 100 lowest-energy docking poses. The ABS values were normalized in order to obtain the NIP value per residue [37]. Residues with NIP value greater or equal to 0.2 were predicted to be interface hot-spot residues. This was previously shown to provide a good compromise between precision and sensitivity, and yielded similar success rates to other predictive methods [37]. The novelty here is that the predicted interface patches were extended by including surface residues (relative accessible surface area rASA > 0.1) within 10 Å distance from the predicted interface hot-spot residues. This method was used to characterize nsSNPs as core, interface or non-interacting surface in complexes with no structural data (S6 Table).

The protein-protein docking benchmark 4.0 [44] was used to test the performance of the above described method to predict extended protein-protein interfaces. We processed all the 176 complexes in the benchmark with our docking-based interface prediction protocol, starting from the structures of the unbound proteins. The predicted extended interface patches were compared to the real interfaces, which were composed of those residues within 10 Å of the partner molecule in the complex structure. Then sensitivity and precision of the method were computed as follows.

$$Sensitivity\left(S\right)=\frac{TruePositives}{TruePositives+FalseNegatives}$$ (3)

$$Precision\left(P\right)=\frac{TruePositives}{TruePostives+FalsePositives}$$ (4)

### Identification of interface pathological mutations at RAS/MAPK cascade

We used our interface prediction method to extend a previous study [24] on 956 RASopathy and cancer missense mutations found in 15 genes of the RAS/MAPK pathway: PTPN11, SOS1, RASA1, NF1, KRAS, HRAS, NRAS, BRAF, RAF1, MAP2K2, MAP2K1, SPRED1, RIT1, SHOC2 and CBL. For the determination of possible pathways affected by the nsSNPs at the interface of the proteins, we used the GO annotation for the functional classification of genes provided by PANTHER database [45].

## Supporting information

S1 Table Statistical analysis of nsSNPs frequencies in the disease-related protein interaction networks. (PDF)

S2 Table Proteins associated to each disease according to OMIM database. (TXT)

S3 Table Proteins associated to the six selected diseases based on OMIM database. All of them have available structure or homology-based model in Interactome3D.
(TXT)

S4 Table Proteins contained in the first layer interaction networks of the six selected diseases. Structural coverage according to Interactome3D (EXP: experimental structure; MDL: Homology model; NS: no structure).
(CSV)

S5 Table Protein complexes forming the first layer interaction networks of the six selected diseases, with their structural coverage. Structural information according to Interactome3D (EXP: experimental structure; MDL: Homology model; MDD: Domain-domain model), or docking. NS: no structure for the subunits.
(CSV)

S6 Table nsSNPs associated to the protein interaction networks of the six selected diseases. They are based on *humsavar*, with their location in the protein according to the real complex structures (Interactome3D) or to the docking predictions (pyDockNIP extended). Many of the nsSNPs are characterized based on both structural and docking data, because they are involved in interactions with available structure and in other ones with no available structure.
(CSV)

## References

[1] Freedman ML, Monteiro ANA, Gayther SA, Coetzee GA, Risch A, Plass C, et al Principles for the post-GWAS functional characterization of cancer risk loci. Nat Genet. 2011;43: 513–518. doi: 10.1038/ng.840 21614091 10.1038/ng.840 PMC3325768

[2] Scott AF, Amberger J, Brylawski B, McKusick VA. OMIM: Online Mendelian Inheritance in Man. Bioinformatics: Databases and Systems. pp. 77–84.

[3] Mailman MD, Feolo M, Jin Y, Kimura M, Tryka K, Bagoutdinov R, et al The NCBI dbGaP database of genotypes and phenotypes. Nat Genet. 2007;39: 1181–1186. doi: 10.1038/ng1007-1181 17898773 10.1038/ng1007-1181 PMC2031016

[4] Baase WA, Liu L, Tronrud DE, Matthews BW. Lessons from the lysozyme of phage T4. Protein Sci. 2010;19: 631–641. doi: 10.1002/pro.344 20095051 10.1002/pro.344 PMC2867005

[5] Guerois R, Nielsen JE, Serrano L. Predicting changes in the stability of proteins and protein complexes: a study of more than 1000 mutations. J Mol Biol. 2002;320: 369–387. doi: 10.1016/S0022-2836(02)00442-4 12079393 10.1016/S0022-2836(02)00442-4

[6] Kuhner S, van Noort V, Betts MJ, Leo-Macias A, Batisse C, Rode M, et al Proteome Organization in a Genome-Reduced Bacterium. Science. 2009;326: 1235–1240. doi: 10.1126/science.1176343 19965468 10.1126/science.1176343

[7] Gavin A-C, Aloy P, Grandi P, Krause R, Boesche M, Marzioch M, et al Proteome survey reveals modularity of the yeast cell machinery. Nature. 2006;440: 631–636. doi: 10.1038/nature04532 16429126 10.1038/nature04532

[8] Yu H, Braun P, Yildirim MA, Lemmens I, Venkatesan K, Sahalie J, et al High-quality binary protein interaction map of the yeast interactome network. Science. 2008;322: 104–110. doi: 10.1126/science.1158684 18719252 10.1126/science.1158684 PMC2746753

[9] Aranda B, Achuthan P, Alam-Faruque Y, Armean I, Bridge A, Derow C, et al The IntAct molecular interaction database in 2010. Nucleic Acids Res. 2010;38: D525–31. doi: 10.1093/nar/gkp878 19850723 10.1093/nar/gkp878 PMC2808934

[10] Szklarczyk D, Franceschini A, Wyder S, Forslund K, Heller D, Huerta-Cepas J, et al STRING v10: protein-protein interaction networks, integrated over the tree of life. Nucleic Acids Res. 2015;43: D447–52. doi: 10.1093/nar/gku1003 25352553 10.1093/nar/gku1003 PMC4383874

[11] Kanehisa M. The KEGG Database. Novartis Foundation Symposia. pp. 91–103. 12539951

[12] Rolland T, Taşan M, Charloteaux B, Pevzner SJ, Zhong Q, Sahni N, et al A proteome-scale map of the human interactome network. Cell. 2014;159: 1212–1226. doi: 10.1016/j.cell.2014.10.050 25416956 10.1016/j.cell.2014.10.050 PMC4266588

[13] Rual J-F, Venkatesan K, Hao T, Hirozane-Kishikawa T, Dricot A, Li N, et al Towards a proteome-scale map of the human protein-protein interaction network. Nature. 2005;437: 1173–1178. doi: 10.1038/nature04209 16189514 10.1038/nature04209

[14] Wu X, Al Hasan M, Chen JY. Pathway and network analysis in proteomics. J Theor Biol. 2014;362: 44–52. doi: 10.1016/j.jtbi.2014.05.031 24911777 10.1016/j.jtbi.2014.05.031 PMC4253643

[15] Zhong Q, Simonis N, Li Q-R, Charloteaux B, Heuze F, Klitgord N, et al Edgetic perturbation models of human inherited disorders. Mol Syst Biol. 2009;5: 321 doi: 10.1038/msb.2009.80 19888216 10.1038/msb.2009.80 PMC2795474

[16] Zanzoni A, Soler-López M, Aloy P. A network medicine approach to human disease. FEBS Lett. 2009;583: 1759–1765. doi: 10.1016/j.febslet.2009.03.001 19269289 10.1016/j.febslet.2009.03.001

[17] David A, Sternberg MJE. The Contribution of Missense Mutations in Core and Rim Residues of Protein-Protein Interfaces to Human Disease. J Mol Biol. 2015;427: 2886–2898. doi: 10.1016/j.jmb.2015.07.004 26173036 10.1016/j.jmb.2015.07.004 PMC4548493

[18] Wang X, Wei X, Thijssen B, Das J, Lipkin SM, Yu H. Three-dimensional reconstruction of protein networks provides insight into human genetic disease. Nat Biotechnol. 2012;30: 159–164. doi: 10.1038/nbt.2106 22252508 10.1038/nbt.2106 PMC3708476

[19] David A, Razali R, Wass MN, Sternberg MJE. Protein-protein interaction sites are hot spots for disease-associated nonsynonymous SNPs. Hum Mutat. 2012;33: 359–363. doi: 10.1002/humu.21656 22072597 10.1002/humu.21656

[20] Mosca R, Tenorio-Laranga J, Olivella R, Alcalde V, Céol A, Soler-López M, et al dSysMap: exploring the edgetic role of disease mutations. Nat Methods. 2015;12: 167–168. doi: 10.1038/nmeth.3289 25719824 10.1038/nmeth.3289

[21] Teng S, Madej T, Panchenko A, Alexov E. Modeling effects of human single nucleotide polymorphisms on protein-protein interactions. Biophys J. 2009;96: 2178–2188. doi: 10.1016/j.bpj.2008.12.3904 19289044 10.1016/j.bpj.2008.12.3904 PMC2717281

[22] Fraser JS, Gross JD, Krogan NJ. From systems to structure: bridging networks and mechanism. Mol Cell. 2013;49: 222–231. doi: 10.1016/j.molcel.2013.01.003 23352243 10.1016/j.molcel.2013.01.003 PMC3558917

[23] Kiel C, Serrano L. Structural data in synthetic biology approaches for studying general design principles of cellular signaling networks. Structure. 2012;20: 1806–1813. doi: 10.1016/j.str.2012.10.002 23141693 10.1016/j.str.2012.10.002

[24] Kiel C, Serrano L. Structure-energy-based predictions and network modelling of RASopathy and cancer missense mutations. Mol Syst Biol. 2014;10: 727 doi: 10.1002/msb.20145092 24803665 10.1002/msb.20145092 PMC4188041

[25] Cheng TMK, Goehring L, Jeffery L, Lu Y-E, Hayles J, Novák B, et al A structural systems biology approach for quantifying the systemic consequences of missense mutations in proteins. PLoS Comput Biol. 2012;8: e1002738 doi: 10.1371/journal.pcbi.1002738 23093928 10.1371/journal.pcbi.1002738 PMC3475653

[26] Adzhubei I, Jordan DM, Sunyaev SR. Predicting Functional Effect of Human Missense Mutations Using PolyPhen-2. Current Protocols in Human Genetics. 2013 pp. 7.20.1–7.20.41. 10.1002/0471142905.hg0720s76 PMC4480630 23315928

[27] Adzhubei IA, Schmidt S, Peshkin L, Ramensky VE, Gerasimova A, Bork P, et al A method and server for predicting damaging missense mutations. Nat Methods. 2010;7: 248–249. doi: 10.1038/nmeth0410-248 20354512 10.1038/nmeth0410-248 PMC2855889

[28] Sim N-L, Kumar P, Hu J, Henikoff S, Schneider G, Ng PC. SIFT web server: predicting effects of amino acid substitutions on proteins. Nucleic Acids Res. 2012;40: W452–7. doi: 10.1093/nar/gks539 22689647 10.1093/nar/gks539 PMC3394338

[29] Berman HM, Battistuz T, Bhat TN, Bluhm WF, Bourne PE, Burkhardt K, et al The Protein Data Bank. Acta Crystallogr D Biol Crystallogr. 2002;58: 899–907. 12037327 10.1107/s0907444902003451

[30] Mosca R, Céol A, Aloy P. Interactome3D: adding structural details to protein networks. Nat Methods. 2013;10: 47–53. doi: 10.1038/nmeth.2289 23399932 10.1038/nmeth.2289

[31] Chen R, Li L, Weng Z. ZDOCK: an initial-stage protein-docking algorithm. Proteins. 2003;52: 80–87. doi: 10.1002/prot.10389 12784371 10.1002/prot.10389

[32] Zhang C, Lai L. SDOCK: a global protein-protein docking program using stepwise force-field potentials. J Comput Chem. 2011;32: 2598–2612. doi: 10.1002/jcc.21839 21618559 10.1002/jcc.21839

[33] Cheng TM-K, Blundell TL, Fernandez-Recio J. pyDock: electrostatics and desolvation for effective scoring of rigid-body protein-protein docking. Proteins. 2007;68: 503–515. doi: 10.1002/prot.21419 17444519 10.1002/prot.21419

[34] Mashiach E, Nussinov R, Wolfson HJ. FiberDock: Flexible induced-fit backbone refinement in molecular docking. Proteins. 2010;78: 1503–1519. doi: 10.1002/prot.22668 20077569 10.1002/prot.22668 PMC4290165

[35] Mosca R, Pons C, Fernández-Recio J, Aloy P. Pushing structural information into the yeast interactome by high-throughput protein docking experiments. PLoS Comput Biol. 2009;5: e1000490 doi: 10.1371/journal.pcbi.1000490 19714207 10.1371/journal.pcbi.1000490 PMC2722787

[36] Clackson T, Wells J. A hot spot of binding energy in a hormone-receptor interface. Science. 1995;267: 383–386. 7529940 10.1126/science.7529940

[37] Grosdidier S, Fernández-Recio J. Identification of hot-spot residues in protein-protein interactions by computational docking. BMC Bioinformatics. 2008;9: 447 doi: 10.1186/1471-2105-9-447 18939967 10.1186/1471-2105-9-447 PMC2579439

[38] Keskin O, Nussinov R. Similar binding sites and different partners: implications to shared proteins in cellular pathways. Structure. 2007;15: 341–354. doi: 10.1016/j.str.2007.01.007 17355869 10.1016/j.str.2007.01.007

[39] Martin J, Lavery R. Arbitrary protein−protein docking targets biologically relevant interfaces. BMC Biophys. 2012;5: 7 doi: 10.1186/2046-1682-5-7 22559010 10.1186/2046-1682-5-7 PMC3441232

[40] Guney E, Menche J, Vidal M, Barábasi A-L. Network-based in silico drug efficacy screening. Nat Commun. 2016;7: 10331 doi: 10.1038/ncomms10331 26831545 10.1038/ncomms10331 PMC4740350

[41] Sahni N, Yi S, Taipale M, Fuxman Bass JI, Coulombe-Huntington J, Yang F, et al Widespread Macromolecular Interaction Perturbations in Human Genetic Disorders. Cell 2015;161: 647–660. doi: 10.1016/j.cell.2015.04.013 25910212 10.1016/j.cell.2015.04.013 PMC4441215

[42] The UniProt Consortium. The Universal Protein Resource (UniProt). Nucleic Acids Res. 2007;36: D190–D195. doi: 10.1093/nar/gkm895 18045787 10.1093/nar/gkm895 PMC2238893

[43] Jackson RM, Gabb HA, Sternberg MJ. Rapid refinement of protein interfaces incorporating solvation: application to the docking problem. J Mol Biol. 1998;276: 265–285. doi: 10.1006/jmbi.1997.1519 9514726 10.1006/jmbi.1997.1519

[44] Hwang H, Vreven T, Janin J, Weng Z. Protein-protein docking benchmark version 4.0. Proteins: Struct Funct Bioinf. 2010;78: 3111–3114. 10.1002/prot.22830 PMC2958056 20806234

[45] Mi H, Lazareva-Ulitsky B, Loo R, Kejariwal A, Vandergriff J, Rabkin S, et al The PANTHER database of protein families, subfamilies, functions and pathways. Nucleic Acids Res. 2005;33: D284–8. doi: 10.1093/nar/gki078 15608197 10.1093/nar/gki078 PMC540032

