# Supplementary material for: Docking-based modeling of protein-protein interfaces for extensive structural and functional characterization of missense mutations
Source: PLoS One. 2017 Aug 25;12(8):e0183643. doi: 10.1371/journal.pone.0183643 (PMC5571915; doi:10.1371/journal.pone.0183643)
Supplement: S1 Table — (PDF) [file pone.0183643.s001.pdf]

S1 Table. Statistical analysis of nsSNPs frequencies in the disease-related protein interaction networks

Type	Region	Odds ratio	CI upper	CI lower	*P*-value
**Only structures**					
Disease	core vs non-core	2.34	2.03	2.69	<0.05
Polymorphism	core vs non-core	0.78	0.63	0.96	<0.05
Unclassified	core vs non-core	1.34	1.04	1.72	<0.05
Disease	interface vs surface	0.94	0.78	1.15	0.56
Polymorphism	interface vs surface	1.33	1.08	1.63	<0.05
Unclassified	interface vs surface	0.80	0.59	1.08	0.14
Disease	core vs interface	2.41	2.02	2.89	<0.05
Polymorphism	core vs interface	0.67	0.53	0.84	<0.05
Unclassified	core vs interface	1.53	1.12	2.10	<0.05
**Only models**					
Disease	core vs non-core	2.05	1.84	2.28	<0.05
Polymorphism	core vs non-core	0.94	0.80	1.09	0.45
Unclassified	core vs non-core	1.21	0.97	1.50	0.08
Disease	interface vs surface	1.42	1.22	1.64	<0.05
Polymorphism	interface vs surface	0.81	0.69	0.96	<0.05
Unclassified	interface vs surface	1.69	1.32	2.16	<0.05
Disease	core vs interface	1.69	1.48	1.93	<0.05
Polymorphism	core vs interface	1.07	0.88	1.29	0.51
Unclassified	core vs interface	0.92	0.72	1.17	0.47

**Structures and models**						
	Disease	core vs non-core	2.06	1.86	2.29	<0.05
	Polymorphism	core vs non-core	0.90	0.77	1.04	0.16
	Unclassified	core vs non-core	1.27	1.04	1.55	<0.05
	Disease	interface vs surface	1.51	1.31	1.74	<0.05
	Polymorphism	interface vs surface	1.08	0.92	1.26	0.33
	Unclassified	interface vs surface	1.58	1.25	2.00	<0.05
	Disease	core vs interface	1.70	1.50	1.92	<0.05
	Polymorphism	core vs interface	0.86	0.72	1.02	0.09
	Unclassified	core vs interface	1.03	0.82	1.29	0.82
